# Enhanced Lightweight Structures Through Brachistochrone-Inspired Lattice Design

**DOI:** 10.3390/polym17050654

**Published:** 2025-02-28

**Authors:** Parisa Majari, Daniel Olvera-Trejo, Jorge A. Estrada-Díaz, Alex Elías-Zúñiga, Oscar Martinez-Romero, Claudia A. Ramírez-Herrera, Imperio Anel Perales-Martínez

**Affiliations:** Tecnologico de Monterrey, Institute of Advanced Materials for Sustainable Manufacturing, Ave. Eugenio Garza Sada 2501, Col: Tecnológico, Monterrey 64700, NL, Mexico; majari@tec.mx (P.M.); j.estrada@tec.mx (J.A.E.-D.); aelias@tec.mx (A.E.-Z.); claudia.ramirezh@tec.mx (C.A.R.-H.); anel.perales@tec.mx (I.A.P.-M.)

**Keywords:** lightweight structures, brachistochrone, lattice, additive manufacturing, polymers, elastomers

## Abstract

Lattice structures offer unique mechanical properties and versatility in engineering applications, yet existing designs often struggle to balance performance and material efficiency. This study introduces the brachistochrone curve as a novel framework for optimizing lattice geometries, enhancing mechanical behavior while minimizing material usage. Using finite element simulations and compressive testing of 3D-printed samples, we analyzed the mechanical response of brachistochrone-based (B-) and standard lattice structures (diamond, IWP, gyroid, and BCC). We investigated the scaling behavior of the volume-to-surface area ratio, incorporated fractal dimension analysis, and compared experimental and numerical results to evaluate the performance of B-lattices versus standard designs (S-). Our findings indicate that brachistochrone-inspired lattices enhance mechanical efficiency, enabling the design of lightweight, high-strength components with sustainable material use. Experimental results suggest that B-gyroid lattices exhibit lower stiffness than S-gyroid lattices under small displacements, highlighting their potential for energy absorption applications.

## 1. Introduction

Additive manufacturing technologies, such as Fused Deposition Modeling (FDM), Selective Laser Melting (SLM), and Stereolithography (SLA), enable the fabrication of complex geometries, creating new opportunities for high-performance and innovative designs. Integrating the brachistochrone curve into lattice structures leverages optimization principles to enhance mechanical properties while reducing material usage—a critical factor for sustainable engineering solutions. The brachistochrone curve, a classical problem in the calculus of variations, defines the optimal path a particle follows under gravity to travel between two points in the shortest time.

Lattice structures are ordered arrangements of repeating complex geometries, valued for their lightweight nature and high surface area, making them essential across various industries. In automotive [1] and heat transfer applications [2], they contribute to weight reduction, lower carbon emissions, and improved fuel efficiency. Their impact absorption capabilities also enhance the performance of lightweight protective gear [3,4,5]. Additionally, in medical applications, lattice structures mimic the mechanical properties of natural bone, improving implant and prosthetic integration for enhanced patient recovery [6,7,8]. By incorporating the brachistochrone curve into lattice design, we aim to advance the development of next-generation lightweight components [9,10,11].

Beyond structural applications, lattice architectures significantly enhance surface area, a key factor in improving the efficiency and performance of advanced functional components. In tissue engineering, they serve as scaffolds for cell growth and regeneration [12], while in catalysis reactors, they optimize reaction kinetics and heat transfer [13]. In energy storage, lattice structures improve battery performance and electrode durability [14,15,16,17]. Similarly, they contribute to the mechanical stability of solar panels [18,19] and enhance desalination technologies by improving filtration efficiency and structural resilience [20,21]. Their high surface area also plays a crucial role in environmental purification and energy storage applications [22]. In biomedical fields, lattice structures support cell growth, tissue regeneration, and targeted drug delivery, making them valuable for regenerative medicine and advanced therapeutic applications [23,24,25,26].

Higuchi’s numerical experiments established a relationship between the power-law index and fractal dimension in time-series spectra [27,28], which can be extended to material properties by analyzing their scaling behavior. The fractal dimension quantifies how completely a fractal fills space as the scale changes, providing a measure of complexity and detail variation across different scales. In this study, we use it to examine the self-similarity and geometric complexity of lattice structures and their influence on mechanical properties, including stress–strain distribution.

Lattice structures exhibit multi-scale behavior, where mechanical properties are governed by interactions at different length scales. Due to their fractal nature, these structures demonstrate isotropic, self-similar, and multi-scale mechanical responses that follow power-law relationships, which can be captured through mathematical fractal expressions. This approach enables the prediction and optimization of mechanical properties across scales, making it crucial for designing materials where nano- and micro-scale characteristics significantly influence macro-scale performance [29,30,31,32].

Numerical simulations using finite element analysis (FEA) provide critical insights into how the fractal nature of lattice structures influences their mechanical properties. By simulating various lattice configurations under different loading conditions, stress–strain distributions can be analyzed to identify optimal designs for specific applications. Understanding the relationship between fractal dimension and mechanical properties enables the development of lightweight, strong, and resilient lattice structures—essential for aerospace, automotive, and biomedical engineering, where high-performance components are required. By leveraging fractal dimensions, isotropy, and power-law scaling, lattice structures can be optimized to create efficient, robust, and application-specific materials [33,34,35].

Previous studies have demonstrated the effectiveness of MATLAB’s Partial Differential Equation (PDE) Toolbox in addressing complex material behaviors and optimizing lattice structures for engineering applications [36,37,38]. In this study, we employ the finite element method (FEM) through the PDE Toolbox to model lattice structures and analyze their stress-displacement behavior. The PDE Toolbox’s capability to define intricate geometries and material properties enables simulations that closely replicate real-world conditions, improving the accuracy of predictive models and aiding in the optimization of lattice-based designs.

This paper is structured as follows: Section 2 examines the power-law relationship between the volume-to-surface area ratio in brachistochrone lattices. Comparisons with standard lattice configurations highlight the impact of geometric complexity, integrating fractal dimension analysis with simulation results to explore correlations with stress-displacement distribution [39,40,41,42,43,44]. Section 3 details the three-dimensional lattice generation process, including STL file creation for 3D printing, and evaluates mechanical performance through compressive testing and numerical simulations using the PDE Toolbox. This section predicts mechanical responses under controlled loading conditions, integrating experimental and computational analyses to provide a comprehensive assessment of lattice behavior. Finally, Section 4 presents the conclusions, summarizing key findings, discussing their implications for lattice structure optimization, and outlining future research directions.

## 2. Brachistochrone-Based TPMS Lattice Structures

The brachistochrone problem seeks the curve along which a particle travels between two points in the shortest time under the influence of gravity. In this section, we adapt the brachistochrone curve for use in lattice structures to explore their properties and mechanical behavior in comparison to regular lattice designs.

The cycloid, the solution to the brachistochrone problem, is defined parametrically as:

(1) x(θ)=r(θ−sinθ),

(2) y(θ)=r(1−cosθ),

where *r* is the radius of the generating circle, and θ is the angle of rotation of the rolling circle. To construct a periodic function, the curve is mirrored across the x-axis, creating a symmetric oscillation. The mirrored curve is given by:

(3) $$x_{m}(\theta )=-r(\theta -\sin\theta ),$$

(4) $$y_{m}(\theta )=-r(1-\cos\theta ).$$

**Figure 1:**
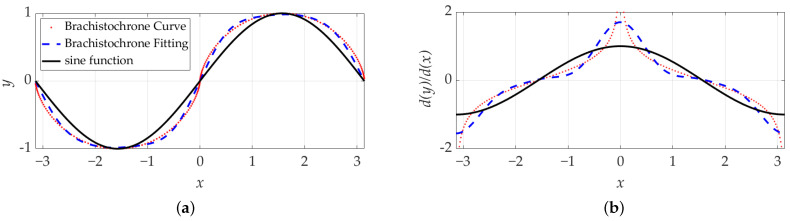
(**a**) Brachistochrone curve and its Fourier fit vs. sin(x) term; (**b**) Derivative of the brachistochrone curve and its Fourier fit vs. the derivative of the sin(x) term.

This transformation produces a continuous and smooth curve that oscillates about the x-axis, forming the basis for periodic lattice designs. Figure 1 illustrates (a) the brachistochrone curve with its Fourier fit compared to the sin(x) term and (b) the derivative of the brachistochrone curve alongside its Fourier fit.

To ensure the successful fabrication of lattice structures without defects or discontinuities, smooth and continuous representation is critical. The Fourier series provides such an approximation by expressing the brachistochrone curve as a sum of sine and cosine functions:

(5) $$f(x)=a_{0}+\sum_{n=1}^{\infty }\left(a_{n}\cos\left(\frac{2\pi nx}{L}\right)+b_{n}\sin\left(\frac{2\pi nx}{L}\right)\right),$$

where *L* is the period of the function, and $a_{n}$ and $b_{n}$ are Fourier coefficients. This formulation enables a smooth, periodic curve suitable for lattice applications.

The Fourier series provides a simplified mathematical representation, making it highly beneficial for computational applications where a continuous function is required to describe a periodic signal. In our study, a fifth-order Fourier series was sufficient to accurately capture the essential features of the brachistochrone curve. The fit was evaluated based on how well the series matched the actual data points, with higher-order terms enhancing precision.

The equation representing the fitness of the brachistochrone curve using a Fourier series is:

(6) $$\begin{array}{c} \sin^{\prime }(x)=b_{1}\sin(x)+b_{2}\sin(2x)+b_{3}\sin(3x)+b_{4}\sin(4x)+b_{5}\sin(5x), \end{array}$$

where the coefficients are:

$$\begin{array}{cc} b_{1} & =1.104,\\ b_{2} & =0.01263,\\ b_{3} & =0.1408,\\ b_{4} & =0.0122,\\ b_{5} & =0.06373. \end{array}$$

The modified cosine terms are given by:

(7) $$\begin{array}{c} \cos^{\prime }(x)=b_{1}\sin\left(x+\frac{\pi }{2}\right)+b_{2}\sin\left(2\left(x+\frac{\pi }{2}\right)\right)+b_{3}\sin\left(3\left(x+\frac{\pi }{2}\right)\right)\\ +b_{4}\sin\left(4\left(x+\frac{\pi }{2}\right)\right)+b_{5}\sin\left(5\left(x+\frac{\pi }{2}\right)\right). \end{array}$$

Using these modified expressions, new lattice structures based on the brachistochrone curve are created by replacing the terms sin(x) and cos(x) in the equations of Triply Periodic Minimal Surfaces in Table 1 with $\sin^{\prime }\left(x\right)$ and $\cos^{\prime }\left(x\right)$, respectively. We designate this modified configuration as the B-lattice, while the standard configuration is referred to as the S-lattice.

Triply Periodic Minimal Surfaces (TPMS) are fascinating geometrical constructs that exhibit intricate periodicity and minimal surface area, making them significant in various fields, including materials science and engineering. Among the prominent TPMS structures are the diamond, gyroid, and Skeletal Interlocking Wall Panel (Skeletal-IWP) configurations. The diamond TPMS is characterized by its cubic symmetry, resembling the atomic arrangement found in diamond crystals, with two interpenetrating networks that provide remarkable mechanical strength and stability, making it an attractive option for designing lightweight yet robust components [45]. In contrast, the gyroid TPMS showcases an intricate, wave-like surface that creates a highly porous structure, allowing for isotropic mechanical properties and making it ideal for applications such as filtration and scaffolds in tissue engineering. Skeletal-IWPs feature smooth, interconnected frameworks that provide superior specific strength and energy absorption compared to traditional porous materials. The main Skeletal-IWP units utilize a body-centered cubic topology, characterized by structural elements that converge at the center of the unit cell, facilitating effective load distribution and mechanical stability under axial forces. In contrast, secondary Skeletal-IWP units are designed with an edge-centered cubic topology, where the structural components are arranged along the symmetry axes of the cube’s faces. This difference in topology leads to distinct mechanical behaviors when subjected to forces, highlighting the importance of studying the cracking responses of both unit types. Investigating these structures not only enhances the understanding of their mechanical properties but also broadens their potential applications in civil engineering, where larger unit sizes are crucial for practical use [46,47,48]. To simplify terminology, we will refer to the secondary Skeletal-IWP as IWP and the main Skeletal-IWP as BCC.

By examining the relationship between the brachistochrone curve and lattice configurations, we can identify the potential advantages of integrating this mathematical framework into lattice design. In this study, we fit the derived brachistochrone curve using a Fourier series to gain deeper insight into its properties and behavior in relation to these lattice structures.

The results presented in Figure 1 illustrate the brachistochrone curve and its Fourier series representation. Figure 1a shows the brachistochrone curve (red) alongside its Fourier fit (blue), demonstrating how accurately the series aligns with the actual data points. Figure 1b presents the derivative of the brachistochrone curve and its Fourier fit compared to the derivative of the sine function. A key observation is that the derivative of the brachistochrone curve (blue line) has a steeper slope than the sine function derivative (black line), particularly at x=0, where the slope of the brachistochrone derivative is much greater than one, while the sine function derivative is equal to one. This difference in derivative behavior could significantly impact the mechanical properties and performance of lattice structures derived from the brachistochrone curve.

Table 2a–d shows the TPMS unit cells for B-diamond, B-gyroid, B-BCC, and B-IWP lattices, each with a size of 30 mm. The differences between S-lattices and their corresponding B-lattices are illustrated in Table 2e–h, where the blue lattice represents the B-configuration and the red lattice represents the S-configuration. The B-lattices exhibit distinct geometric structures compared to traditional S-lattices, suggesting potential differences in mechanical properties and performance. For instance, the B-diamond lattice shows thin-film variations in its surface structure, while the B-BCC lattice has a larger volume than the standard configuration.

Building on this foundation, the next section explores the computational analysis of these newly derived lattices. We evaluated geometric properties such as volume, surface area, and fractal dimension using advanced mathematical tools and simulations. These analyses are critical for understanding the mechanical behavior and potential applications of these innovative structures. Our study focuses on both B- and S-lattice geometries, including gyroid, IWP, BCC, and diamond configurations.

### 2.1. Fractal Dimension

By employing the box-counting method, we calculated the fractal dimension ($D_{f}$), which provides insights into the complexity and scaling behavior of TPMS structures. The fractal dimension quantifies how the structural complexity scales with size and is defined as:

(8) $$D_{f}=-\lim_{\epsilon \to 0}\frac{\log(N(\epsilon ))}{\log(\epsilon )},$$

where N(ϵ) is the number of boxes required to cover the fractal, and ϵ is the size of the boxes [49]. In our implementation, $D_{f}$ is calculated using:

(9) $$D_{f}=\frac{\log\left(\sum (OBJ(:)>0)\right)}{\log\left(\frac{1}{\epsilon }\right)},$$

where ∑(OBJ(:)>0) counts the positive voxels corresponding to the number of occupied boxes, and ϵ represents the size of the box. This logarithmic ratio estimates the fractal dimension.

**Figure 2:**
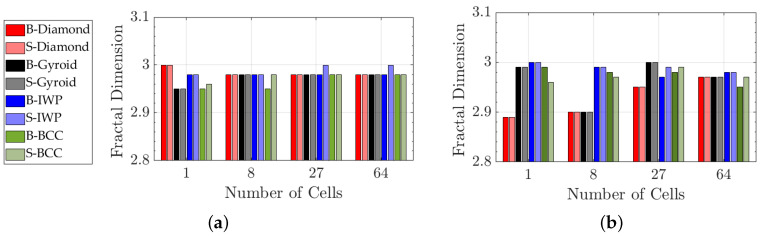
Fractal dimension as a function of number of cells for B- and S-diamond, IWP, gyroid, BCC. (**a**) Length of unit cell is equal to 3 mm; (**b**) the length of lattice equal is equal to 30 mm.

Figure 2 illustrates the relationship between the fractal dimension and the number of cells for B- and S-diamond, IWP, gyroid, and BCC lattice structures. In Figure 2a, the unit cell length is fixed at 3 mm, and box sizes of ϵ=0.037mm,0.03mm, and 0.25mm were used to balance computational accuracy and efficiency. The results show that the fractal dimension remains relatively constant as the number of cells increases across all lattice types.

In Figure 2b, the lattice length is set to 30 mm, and similar trends are observed. Increasing the number of cells while maintaining the geometric arrangement does not significantly alter the fractal dimension. This indicates that $D_{f}$ is a robust and scale-invariant property of these lattice structures, determined primarily by their intrinsic geometry rather than the number of repeating units.

The fractal dimension serves as a reliable descriptor of the complexity and space-filling properties of lattice architectures. Its scale-invariance implies that it reflects the self-similarity of the pattern at different scales, making it an effective tool for characterizing lattice structures regardless of their overall size.

### 2.2. Ratio Volume to Surface Area

**Figure 3:**
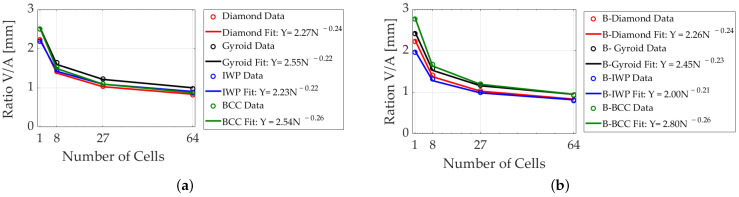
Power-law relationship of the volume-to-surface area ratio as a function of the number of cells, based on equations in Table 1 with C=0 and $\epsilon =L_{u}/90$. The lattice size is fixed at 30mm for configurations with 1, 8, 27, and 64 cells, corresponding to unit sizes of 30mm, 15mm, 10mm, and 7.5mm, respectively. (**a**) S-lattices: diamond, IWP, gyroid, and BCC. (**b**) B-lattices: diamond, IWP, gyroid, and BCC.

Figure 3 illustrates the power-law relationship between the ratio of volume to surface area and the number of cells for different lattice structures, with the lattice length fixed. This comparison includes B- and S-lattices for diamond, IWP, gyroid, and BCC structures. As the number of cells increases, this ratio decreases across all structures, following a predictable power law.

Both B- and S-lattices adhere to this power law but exhibit different parameters. By comparing their fit lines, we observe how B-structures influence thickness differently than S-geometries. These trends are crucial for designing lattice structures with specific mechanical properties, as they allow for predictions about how changes in cell number affect thickness, strength, and flexibility. Notably, the power-law fits for the standard lattices—diamond, IWP, gyroid, and BCC—show differences from their B-based counterparts, except for the diamond lattice, where the power-law fit remains consistent between S- and B-lattices.

Understanding the fractal dimension is essential, as it directly relates to the geometric properties of TPMS structures, such as their surface area. The total surface area of a TPMS structure is computed by iterating through each triangular face in the isosurface representation. For each triangular face, the area is calculated using its vertices:

(10) $$\mathrm{area}=\frac{1}{2}∥v_{2}-v_{1}∥\times ∥v_{3}-v_{1}∥,$$

where $v_{1},v_{2},v_{3}$ are the coordinates of the triangle vertices. The total surface area is then obtained by summing the areas of all triangular faces:

(11) surface_area=∑area.

**Figure 4:**
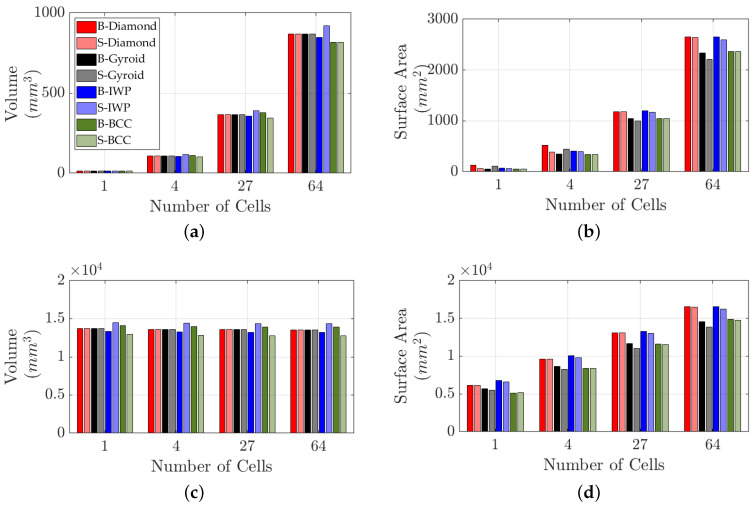
Volume and surface area as functions of the number of cells for B- and S-lattices: diamond, IWP, gyroid, and BCC. (**a**,**b**) correspond to a unit cell length of 3mm for configurations with 1, 8, 27, and 64 cells, with lattice sizes of 1, 2, 3 and 4mm. (**c**,**d**) correspond to lattices with 1, 8, 27, and 64 cells, with unit cell lengths of 30, 15, 10 and 7.5 mm, respectively.

This parameter is critical for understanding how TPMS structures interact with their environment, particularly in applications such as filtration and catalysis. Figure 4 investigates the surface area and volume of diamond, IWP, gyroid, and BCC lattice structures as a function of the number of cells. Figure 4a,b focus on scenarios where the length of the unit cell remains constant across all lattice types, while Figure 4c,d explore cases where the length of the lattice is fixed but the lengths of the unit cells vary. In Figure 4b,d, the surface area increases with the number of cells, with B-lattices consistently showing equal or larger surface areas compared to their S-lattice counterparts. Notably, B-diamond and B-IWP lattices have the largest surface areas.

Figure 4a,c present the volume of the lattices. When the unit cell length is constant, the volume increases linearly with the number of cells. However, when the lattice length is fixed, the volume remains relatively stable across varying unit cell sizes [50,51,52]. Notably, B- and S-diamond, as well as gyroid lattices, exhibit identical volumes under these conditions. In contrast, S-IWP has the maximum volume, resulting in the lowest density, while S-BCC has the minimum volume, leading to the highest density.

As shown in Figure 4a,c, the volume difference between the B-BCC and S-BCC lattices, as well as between B-IWP and S-IWP lattices, is evident. The volume of a lattice structure is primarily influenced by its geometry size, while the fractal dimension (Figure 2) is an intrinsic property of the mathematical expression defining the lattice structure. Volume, as a scale-dependent property, is directly influenced by the overall shape and spatial arrangement of the geometric forms. Different geometries naturally lead to variations in volume, even when the number of cells remains constant. In contrast, the fractal dimension represents the fundamental, scale-invariant characteristics of the structure as defined by its mathematical expression. Since this expression remains unchanged regardless of the number of cells, the fractal dimension remains relatively stable, capturing the intrinsic self-similar and space-filling nature of the lattice. This distinction explains why volume, which is scale-dependent, is influenced by specific geometric and topological variations. In contrast, the fractal dimension serves as a more fundamental descriptor of the structural complexity of the lattice.

**Figure 5:**
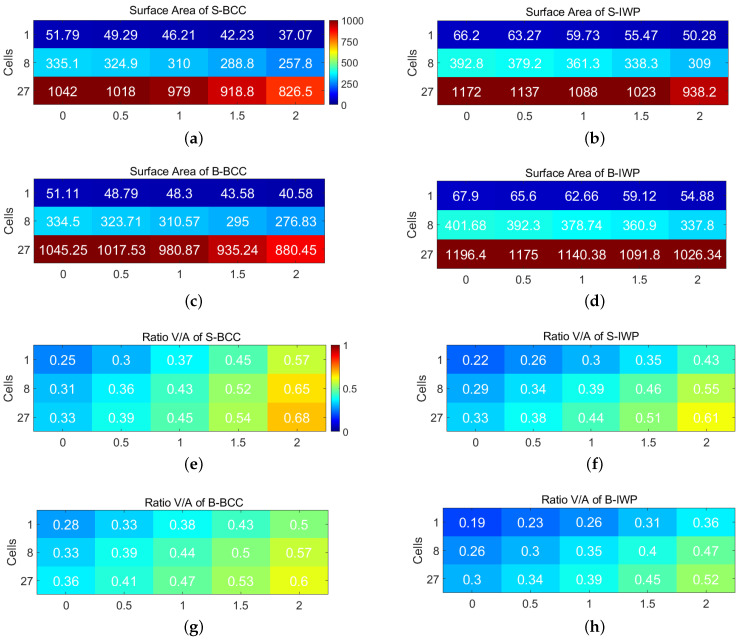
Surface area and ratio of volume to surface area as functions of the number of cells and constant *C* for S-lattices with ϵ=0.075. (**a**) Surface area of BCC. (**b**) Surface area of IWP. (**c**) Ratio of volume to surface area for BCC. (**d**) Ratio of volume to surface area for IWP. (**e**) Surface area of B-BCC. (**f**) Surface area of B-IWP. (**g**) Ratio of volume to surface area for B-BCC. (**h**) Ratio of volume to surface area for B-IWP. Unit cell size is fixed at 3mm.

To further analyze geometric properties, Figure 5 focuses on BCC and IWP lattice structures with a fixed unit cell size of 3mm. This figure provides a detailed comparison of surface area and the ratio of volume to surface area for these lattices as functions of the number of cells and a constant *C*, with a fixed lattice parameter of ϵ=0.075.

Figure 5a,b illustrate the surface area for BCC and IWP. The surface area increases with the number of cells, and IWP consistently exhibited larger surface areas than BCC. This trend highlights the impact of IWP’s geometric configuration on surface area, which may influence its physical properties. Additionally, increasing *C* reduces the surface area for both lattices.

Figure 5c,d examine the volume to surface area ratio, providing insights into structural efficiency. This ratio increases with the number of cells and *C*, emphasizing how geometric parameters affect the performance characteristics of these lattice structures.

## 3. Experimental Measurements and Numerical Results

This section outlines the computational approach used for the generation of STL files and manufacturing of lattice structures, detailing the MATLAB-based workflow for creating intricate geometries suitable for additive SLA manufacturing.

### 3.1. Manufacturing of Lattice Structures for Compressive Test

First, the lattice structures were generated using a MATLAB R2024b (MathWorks Inc., Natick, NA, USA) script that computes the geometric configurations of the B- and S- lattice types. The process involved defining a mesh grid and calculating isosurfaces for the specified lattice equations listed in Table 1. The *isosurface* and *isocaps* functions were used to extract 3D isosurfaces from the computed lattice data, creating a mesh that accurately represents each lattice structure. These functions were applied to both B- and S- lattices to generate the corresponding STL files. The resulting meshes were exported to STL format using the *stlwrite* function, ensuring compatibility with most 3D printers. This approach allowed precise control over geometric parameters, enabling the generation of detailed STL files for each lattice type. The flexibility of this framework facilitates the exploration of various lattice configurations while ensuring reproducibility.

**Figure 6:**
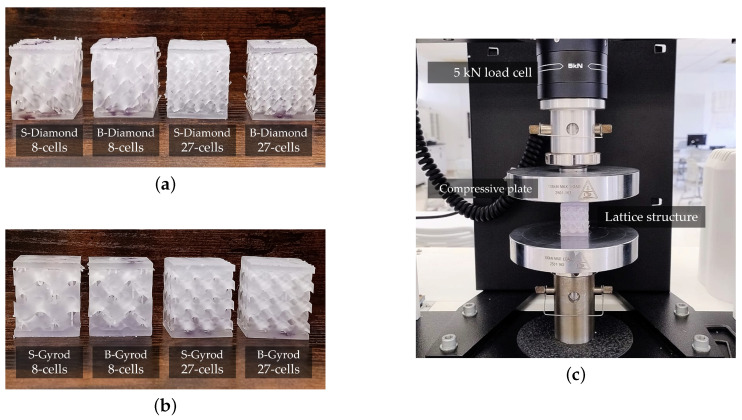
(**a**) B- and S-diamond printed lattices with 8 and 27 cells, (**b**) B- and S- gyroid printed lattices with 8 and 27 cells, (**c**) compressive tests using a Uniaxial Test Machine.

The STL files were processed using PreForm 3.44.2 software with Elastic 50A Resin V1 for additive manufacturing on a FormLabs Form 3 printer. The lattices were printed using the recommended parameters: 100 µm layer thickness and 1 s exposure time. Post-processing involved washing the printed lattices in an isopropyl alcohol bath for 10 min, followed by post-curing at 60 °C for another 10 min. Diamond and gyroid S- and B-lattices with 8- and 27-cell configurations were fabricated. Figure 6 presents the 3D-printed samples of B- and S-diamond and gyroid lattice structures, along with the experimental setup. Figure 6a displays the B-diamond and S-diamond lattices with 8 and 27 cells, while Figure 6b shows the B-gyroid and S-gyroid lattices with the same configurations. These samples were subjected to compressive tests using a Universal Testing Machine (Instron, Norwood, MA, USA) at a speed of 5 mm/min to obtain force-versus-displacement data. Figure 6c illustrates the compressive testing setup for the printed lattice samples using the Universal Testing Machine. The experimental results were later compared with simulation data to assess the performance and accuracy of the computational models.

**Figure 7:**
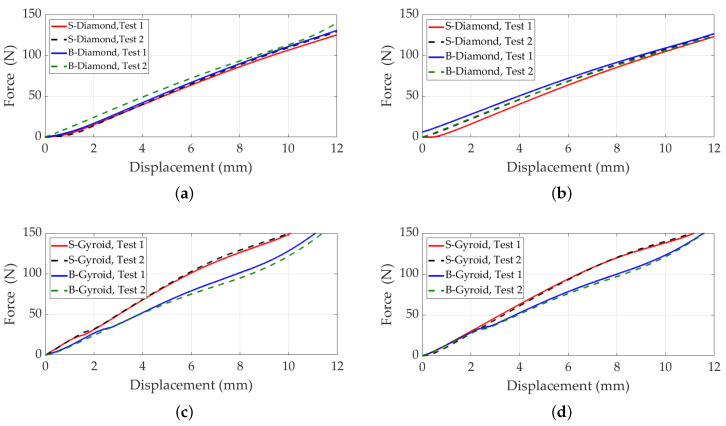
Displacement versus compressive force for S- and B-diamond and gyroid lattices with C=0. The force was applied to the upper faces in the *z*-direction, while the lower faces were fixed. (**a**) S- and B-diamond lattices with 8 cells, unit cell size 15mm. (**b**) S- and B-diamond lattices with 27 cells, unit cell size 10mm. (**c**) S- and B-gyroid lattices with 8 cells, unit cell size 15mm. (**d**) S- and B-gyroid lattices with 27 cells, unit cell size 10mm.

Figure 7 presents the displacement versus compressive force results for S- and B-diamond and gyroid lattices. As observed, there is no significant difference between the B- and S-diamond lattices, consistent with our earlier findings showing that the power-law fit for standard and B-diamond lattices remains consistent when lattice length is fixed. The power-law fit accurately captures the mechanical properties of these lattices, providing reliable predictions and facilitating comparisons. Figure 7c,d present the displacement versus compressive force results for S- and B-gyroid lattice structures, with C=0. The force was applied to the upper faces in the *z*-direction, while the lower faces were fixed. These results were obtained from experimental tests conducted on three printed samples of each configuration.

The results indicate that increasing the number of cells and reducing the cell size improves the mechanical performance of the gyroid lattice, as evidenced by a decrease in the required compressive force. Specifically, the 27-cell configuration exhibits a smaller compressive force compared to the 8-cell configuration, highlighting the benefits of increased cell density. A comparison between B- and S-gyroid lattices reveals notable differences in mechanical properties due to geometric variations. The compressive force at certain displacements is greater for the S-gyroid compared to the B-gyroid. This difference is attributed to the geometric alterations in the B-gyroid, which influence how the structure distributes and withstands applied forces. The S-gyroid’s higher compressive force indicates greater stiffness, making it suitable for structural applications requiring rigidity and resistance to deformation, such as supporting heavy loads. Conversely, the B-gyroid, with lower compressive forces at equivalent displacements, offers greater flexibility and energy absorption. This makes it advantageous for applications requiring impact resistance or shock absorption, such as protective components or cushioning systems. Additionally, the altered geometry of the B-gyroid contributes to potential weight reduction, which is beneficial in transportation industries like aerospace and automotive, where minimizing weight is critical.

### 3.2. Numerical Simulation via Finite Element Method

In this study, we utilized the MATLAB Partial Differential Equation (PDE) Toolbox to analyze the mechanical behavior of lattice structures under applied displacement. The primary objective was to compute displacement and Von Mises stress in response to specific loading conditions. The PDE Toolbox enables precise modeling of stress and displacement distributions, allowing us to simulate real-world conditions. By restricting the analysis to small displacements, the material’s response can be accurately described by a linear elastic constitutive model and avoiding the need to capture complex nonlinear behavior for small displacement assumption. Boundary conditions consistent with the experimental setup were applied, with displacement imposed on the upper faces and the lower faces fixed. This approach facilitated controlled predictions of mechanical responses under defined loading scenarios. Elastic resin 50A was selected as the material for this study, with mechanical properties characterized by a Young’s modulus of 2.6 MPa and a Poisson’s ratio of 0.4 [53]. The Von Mises stress was used as a theoretical metric to assess mechanical performance under compressive loading. Although primarily associated with metals, this metric is applicable to polymers like elastic resin 50A, particularly when they exhibit ductile characteristics. Evaluating Von Mises stress provided critical insights into the yield behavior of the material, informing design decisions and material selection [54,55,56]. The simulation process began with importing 3D models of S- and B-diamond structures consisting of 8 and 27 cells, with cell sizes of 15 mm and 10 mm, respectively. The PDE Toolbox allowed for defining complex geometries and material properties, facilitating the generation of finite element meshes composed of quadratic tetrahedral elements. These elements are particularly effective for 3D analyses, as they adapt to intricate geometries and provide accurate representations of structural characteristics. A high-resolution quadratic element formulation was used to ensure mesh quality and result accuracy.

The choice of element type and mesh density in finite element analysis (FEA) significantly influences the accuracy of stress distribution predictions in lattices. Furthermore, mesh density plays a crucial role in enhancing accuracy; however, the findings indicate that increasing the resolution beyond a certain threshold—specifically, using a mesh size of $L_{u}/90$, where $L_{u}$ is the length of the unit cell—does not result in significant improvements in predictive accuracy for small displacements, as nonlinear effects are suppressed in this regime [57].

After mesh generation, a FEM model was employed to solve the governing Partial Differential Equations for structural mechanics. The simulations computed deformation, stress, and strain distributions within the lattice structures under compressive displacement applied in the negative *z*-direction to the upper surfaces. These results provided valuable insights into the mechanical performance of B- and S-lattices, highlighting the distinct behaviors resulting from their geometric configurations.

#### 3.2.1. Diamond Lattice Structures

**Figure 8:**
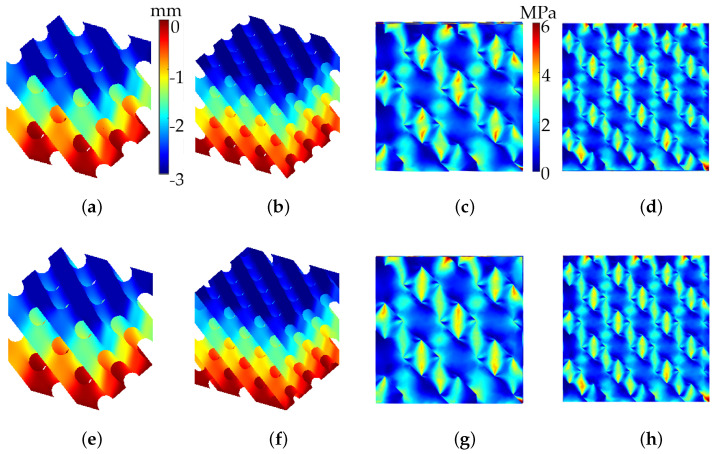
Von Mises stress and *z*-displacement for B-diamond (upper row) and S-diamond (lower row) lattices. The models consist of two configurations: 8 cells (15 mm each, ϵ=0.36mm) and 27 cells (10 mm each, ϵ=0.24mm). The analysis assumes C=0, a *z*-displacement of −3mm, a Young’s modulus of 2.6MPa, and a Poisson’s ratio of 0.4. (**a**,**b**) *z*-displacement for B-diamond lattices; (**c**,**d**) Von Mises stress for B-diamond lattices; (**e**,**f**) *z*-displacement for S-diamond lattices; (**g**,**h**) Von Mises stress for S-diamond lattices.

Given the substantial surface area of the diamond lattice, we began by examining the simulation and experimental analysis of mechanical behavior in B- and S-diamond lattice structures. Figure 8 presents the mechanical behavior of B- and S-diamond lattice structures under applied displacement. Figure 8a,b display the *z*-direction displacement for B-diamond lattices with 8 and 27 cells, respectively. The color gradient indicates the magnitude of displacement, revealing deformation patterns under load. Figure 8c,d illustrate the Von Mises stress distribution for these B-diamond lattices, with zoomed views of the xz-plane, highlighting areas of high stress concentration.

In the lower row, Figure 8e,f show the *z*-direction displacement for S-diamond lattices with 8 and 27 cells, while Figure 8g,h depict their Von Mises stress distribution. The comparison reveals differences in displacement and stress patterns between B- and S-diamond lattices, emphasizing the impact of geometric variations. The number of cells (8 vs. 27) influences both displacement and stress distribution, significantly affecting the mechanical response under identical loading conditions.

**Figure 9:**
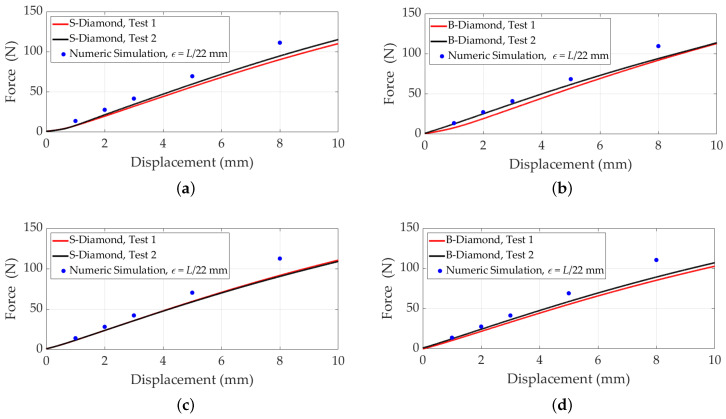
Comparison of simulated and experimental data for displacement versus compressive force for B- and S-diamond lattices with C=0, a Young’s modulus of 2.6MPa, and a Poisson’s ratio of 0.4. A force is applied to the upper faces in the *z*-direction, while the lower faces are fixed. (**a**,**b**) The 8-cell configurations for S-diamond and B-diamond, respectively. (**c**,**d**) The 27-cell configurations for S-diamond and B-diamond, respectively.

To validate the simulation results, Figure 9 compares displacement versus compressive force for B- and S-diamond lattices. The comparison evaluates the accuracy of the simulation model in predicting mechanical behavior under load. By analyzing relative errors at various displacements for configurations with 8 and 27 cells, the alignment between simulated and experimental data is assessed. The comparison indicates that both lattice types exhibit similar mechanical behavior, with simulations closely aligning with experimental results, particularly for 27-cell configurations. This analysis underscores the reliability of the simulation methodology and provides valuable insights into the mechanical performance of these structures for practical applications.

#### 3.2.2. Gyroid Lattice Structures

In this section, we extend our analysis to gyroid lattice structures, comparing B-gyroid and S-gyroid lattices. As depicted in Figure 4, B-gyroid lattices exhibit a larger surface area compared to their S-gyroid counterparts, suggesting potential differences in mechanical performance due to their distinct geometric configurations. To explore these differences, we conducted a comprehensive analysis of the mechanical behavior of gyroid structures under applied loads. This included both simulation and experimental approaches to evaluate the impact of the brachistochrone transformation on gyroid lattice performance. Simulations were performed to compute displacement and Von Mises stress for both configurations, using geometric models consistent with previous analyses. Experimental validation was carried out using 3D-printed gyroid samples fabricated with Elastic 50A resin and processed under identical conditions to the diamond lattices. Compressive tests measured the force-displacement response of the gyroid structures, enabling a direct comparison between B- and S-gyroid configurations.

**Figure 10:**
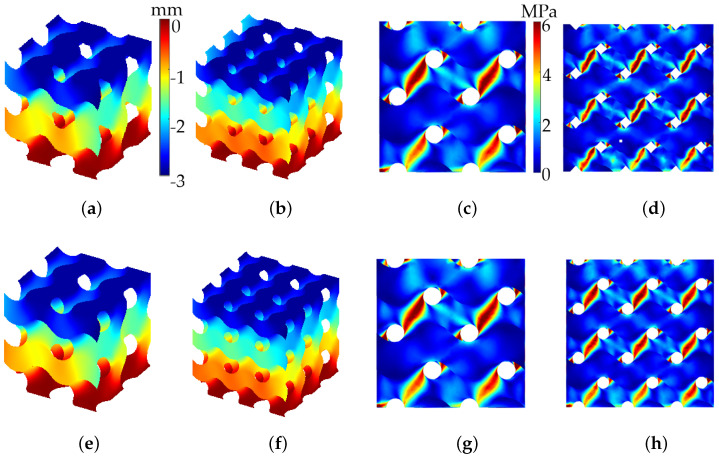
Numerical simulation results for B-gyroid (**top** row, **a**–**d**) and S-gyroid (**bottom** row, **e**–**h**) lattices. The study includes two configurations: 8 cells (15 mm each, ϵ=0.36mm) and 27 cells (10 mm each, ϵ=0.24mm). The analysis assumes C=0, *z*-displacement of −3mm, a Young’s modulus of 2.6MPa, and a Poisson’s ratio of 0.4. Results are presented as *z*-displacement for (**a**,**e**) 8 cells and (**b**,**f**) 27 cells; Von Mises stress for (**c**,**g**) 8 cells and (**d**,**h**) 27 cells.

Figure 10 displays the results of numerical simulations for B-gyroid (top row) and S-gyroid (bottom row) lattice structures. Figure 10a,b show *z*-displacement for B-gyroid lattices with 8 and 27 cells, respectively, with a color gradient indicating deformation under load. Figure 10c,d present the Von Mises stress distribution for these configurations, highlighting high-stress concentration areas. Similarly, Figure 10e,f illustrate *z*-displacement for S-gyroid lattices with 8 and 27 cells, while Figure 10g,h display their Von Mises stress distribution. Observations reveal that B-gyroid lattices exhibit different deformation and stress distribution patterns compared to S-gyroid lattices, likely due to geometric variations. These differences highlight the potential for tailored mechanical properties in B-lattice designs.

**Figure 11:**
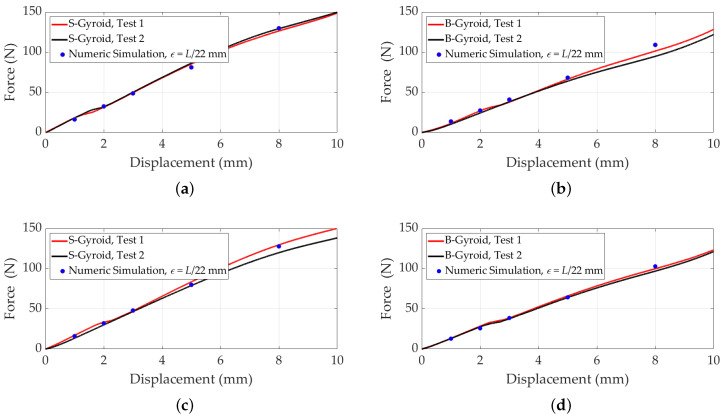
Comparison of simulated and experimental data for displacement versus compressive force for B- and S-gyroid lattices, with C=0, a Young’s modulus of 2.6MPa, and a Poisson’s ratio of 0.4. A force is applied to the upper faces in the *z*-direction, while the lower faces are fixed. (**a**,**b**) 8-cell configurations for S-gyroid and B-gyroid, respectively; (**c**,**d**) 27-cell configurations for S-gyroid and B-gyroid, respectively.

Figure 11 compares displacement versus compressive force for B- and S-gyroid lattice structures, incorporating both experimental and simulation data. The close alignment between simulation and experimental results demonstrates the accuracy of the computational model in predicting the mechanical behavior of gyroid lattices. This efficiency is crucial for optimizing lattice designs, enabling reliable predictions of mechanical properties without extensive physical testing. By capturing key characteristics of B- and S-gyroid configurations, the simulation provides a valuable tool for material design, saving time and resources.

#### 3.2.3. Results Discussion

Comparing experimental data and numerical simulations for S- and B-diamond and gyroid lattices, we observe no significant difference between B- and S-diamond lattices. This finding aligns with the power-law fit for both standard and B-diamond lattices when the lattice length was fixed. The power-law model accurately captures the geometrical properties of these structures. For S- and B-gyroid lattices, displacement versus compressive force data was obtained with the force applied to the upper faces in the z-direction while the lower faces were fixed. The results indicate that increasing the number of cells (i.e., reducing cell size while maintaining a fixed equivalent volume of 30 × 30 × 30 mm) enhances the mechanical performance of gyroid lattices, as a higher compressive force is required to achieve a specific displacement. A direct comparison between B- and S-gyroid lattices reveals notable differences in mechanical properties due to geometric variations. In particular, for displacements ranging from 4 to 10 mm, the S-gyroid lattice exhibits higher compressive force than the B-gyroid, indicating greater stiffness. This suggests that S-gyroid lattices are better suited for structural applications requiring rigidity and resistance to deformation, such as load-bearing components. In contrast, the B-gyroid lattice, which experiences lower compressive forces at equivalent displacements, provides greater flexibility and energy absorption, making it ideal for impact-resistant applications such as protective gear or cushioning systems. Furthermore, the altered geometry of the B-gyroid contributes to a possible weight reduction, which is beneficial in various engineering applications.

**Figure 12:**
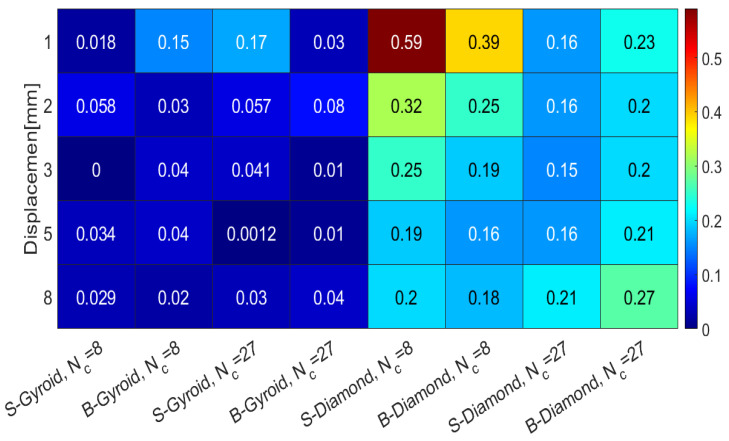
Relative error for B- and S-diamond and gyroid lattices with 8 and 27 cells. The error represents the difference between experimental averages and finite element simulation results.

To further assess the accuracy of the numerical simulations, Figure 12 presents the relative error values between experimental averages and finite element simulation results. Notably, the gyroid lattices exhibit lower relative error than the diamond lattices in both 8- and 27-cell configurations, indicating better agreement between experimental and simulation results.

Manufacturing imperfections, such as layers misalignment or voids, can occur during the 3D printing process of the lattice structures. These imperfections can affect their mechanical behavior under both quasi-static and dynamic loads, leading to differences between the experimental and numerical results. The presence of manufacturing defects can introduce local stress concentrations, alter the load transfer mechanisms within the lattice, and change the overall deformation and failure modes of the structure. Therefore, the error between the experimental and numerical results on the mechanical performance of the 3D-printed lattices could be partially attributed to the influence of manufacturing imperfections.

**Figure 13:**
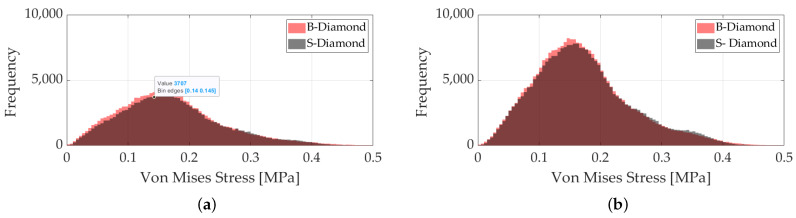

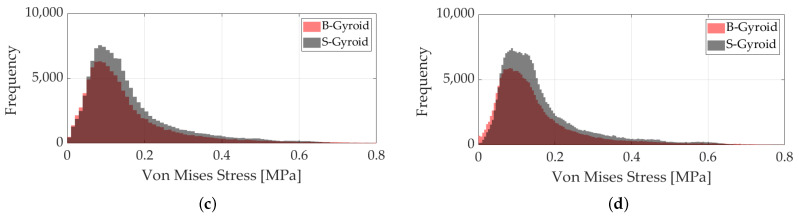
Histograms of Von Mises stress obtained from simulations with C=0, Young’s modulus 2.6MPa, and Poisson’s ratio 0.4. A *z*-displacement of −3mm is applied to the upper faces, with the lower faces fixed. (**a**) S- and B-diamond lattices with 8 cells ($L_{u}=15\;\mathrm{mm}$). (**b**) S- and B-diamond lattices with 27 cells ($L_{u}=10\;\mathrm{mm}$). (**c**) S- and B-gyroid lattices with 8 cells ($L_{u}=15\;\mathrm{mm}$). (**d**) S- and B-gyroid lattices with 27 cells ($L_{u}=10\;\mathrm{mm}$).

To further understand the impact of geometry on mechanical performance, we analyzed the Von Mises stress distribution for B- and S-diamond and gyroid lattices with 8 and 27 cells, maintaining a constant lattice length of 30 mm. Figure 13 presents histograms of Von Mises stress values for these structures under a *z*-displacement of −3mm. The histograms reveal distinct stress distributions for each configuration, highlighting the influence of geometry and cell size on mechanical behavior. For the diamond lattices, the histograms show a clear peak around 0.1–0.2 MPa for both 8- and 27-cell configurations, indicating that most Von Mises stress values fall within this range. In contrast, the gyroid lattices display peaks around 0.05–0.15 MPa, with extended tails indicating a higher frequency of regions experiencing very high stress levels. The differences in stress distribution have significant implications for mechanical performance. The diamond structures exhibit shorter tail distributions, suggesting fewer regions with high stress concentrations and a lower likelihood of localized failure. Conversely, the gyroid structures show longer and more pronounced tails, indicating a greater risk of high-stress regions that could lead to material failure under certain loading conditions. The choice of structural design also impacts stress concentration and distribution. The B-diamond structure exhibits a broader range of Von Mises stress values compared to the S-diamond structure, as seen in Figure 13a,b. Similarly, the B-gyroid structures show distinct stress distribution patterns compared to their S-gyroid counterparts, as illustrated in Figure 13c,d. These results suggest that B-structures have potential advantages in applications requiring tailored stress responses. The differences between the 8- and 27-cell configurations further highlight the role of cell size in mechanical behavior. Smaller cell sizes (27-cell configuration) result in more uniform stress distributions, reducing localized stress concentrations. However, this comes at the cost of slightly higher stress magnitudes in certain regions. Understanding these trade-offs is critical for selecting the appropriate lattice structure for specific applications, where minimizing high-stress regions and ensuring long-term performance are key considerations.

## 4. Conclusions

This study highlights the potential of utilizing the brachistochrone curve in lattice structure design, offering advancements in mechanical performance and material efficiency. The B-lattice exhibits distinct advantages, including increased surface area and altered stress distributions, making it suitable for diverse applications.

The brachistochrone curve introduces subtle changes in the surface topology and curvature of the lattice structures compared to their standard counterparts. No buckling was observed due to the introduction of the brachistochrone curve. Nonetheless, compressive tests were performed under small displacements in the elastic regime and further research may explore the mechanical stability of these novel architectures.

The larger surface area of B-lattices enhances functionalities in several potential engineering applications such as catalysis, energy storage, and thermal management, facilitating faster reaction rates, improved charge–discharge kinetics, and efficient heat dissipation. In filtration and separation processes, B-lattices improve particle filtration and throughput, addressing challenges in clean water and air purification. Furthermore, their potential for supporting cell growth in tissue engineering scaffolds makes them valuable in regenerative medicine.

Comparisons of Von Mises stress revealed that B-lattices redistribute stress differently than standard lattices, suggesting applications in impact resistance and energy absorption. While B- and S-diamond lattices show similar mechanical behavior under compressive loads, B-gyroid lattices demonstrate enhanced flexibility and energy absorption, contrasting with the stiffness of S-gyroid lattices, which is advantageous for load-bearing applications.

The findings also reveal key differences in lattice structures like IWP and BCC. The IWP’s low density make it ideal for aerospace and automotive industries, while the BCC’s high density provides robustness for environments demanding durability. The power law fit for both B- and S-lattices ensures accurate predictions, aiding in material optimization. While this work offers significant insights into the mechanical properties of B-lattices, future research should address nonlinear material behavior and large displacement effects to improve simulation accuracy and expand their applicability in advanced engineering applications.

While this study provides valuable insights into B-lattice mechanical performance, further exploration is needed to understand the effects of geometric perturbations, non-uniform cell distributions, and functional material gradients on stress localization. Currently, no parametric models predict these influences, and numerical simulations, though useful, require fine mesh refinement and nonlinear constitutive modeling, making them computationally demanding. Experimental techniques with Digital Image Correlation (DIC), help capture strain and stress distributions but are limited to exposed surfaces. Future research should integrate experimental, mathematical modeling and computational approaches to develop predictive models that account for both non-uniform lattice structures and material property gradients. Additionally, future studies should explore the effects of oblique loading conditions, the role of symmetry in anisotropic deformation, and geometric modifications at lattice–cell boundaries to mitigate localized stress concentrations and enhance mechanical performance. The introduction of functional material gradients, such as spatially varying Young’s modulus or Poisson’s ratio, could provide tailored mechanical responses and optimize structural efficiency. Investigating the impact of manufacturing imperfections, including layer misalignments and voids, is also crucial for understanding real-world performance deviations. Lastly, incorporating advanced material models, such as plasticity and hyperelasticity, will improve the accuracy of predictions in high-strain rate conditions and large deformation scenarios. These future research directions will be instrumental in refining the design and optimization of B-lattices, expanding their applicability to multi-functional engineering structures, impact-resistant components, and functionally graded materials.

## Figures and Tables

**Table 1:** Equations for various Triply Periodic Minimal Surfaces (TPMS).

Lattice	Equation
Diamond	sin(x)sin(y)sin(z)+sin(x)cos(y)cos(z)+cos(x)sin(y)cos(z)+cos(x)cos(y)sin(z)=C
Gyroid	cos(x)sin(y)+cos(y)sin(z)+cos(z)sin(x)=C
BCC	cos(2x)+cos(2y)+cos(2z)−2[cos(x)cos(y)+cos(y)cos(z)+cos(z)cos(x)]=C
IWP	2[cos(x)cos(y)+cos(y)cos(z)+cos(z)cos(x)]−[cos(2x)+cos(2y)+cos(2z)]=C

**Table 2:** Blue solid representations of unit cells for B-diamond, B-gyroid, B-IWP, and B-BCC, along with the purple STL models obtained by subtracting the S-lattice from their respective B-lattices. All unit cells are 30 mm in size, with tick marks every 10 mm.

	Diamond	Gyroid	IWP	BCC
B-lattice	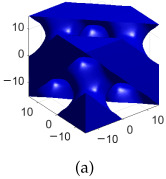	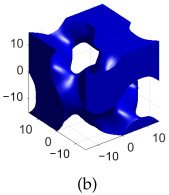	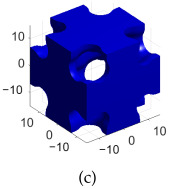	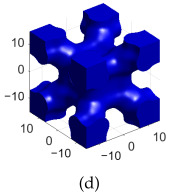
B- subs S- lattice	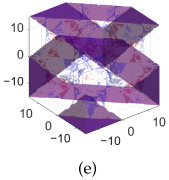	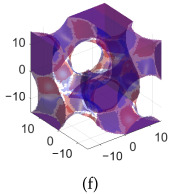	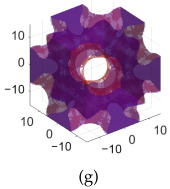	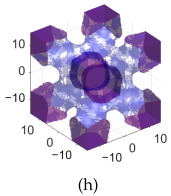

## Data Availability

Data generated or analyzed during this study are available from the corresponding author upon reasonable request. Data available under certain conditions.

## References

[1] Aslan B., Yıldız A.R. (2020). Optimum design of automobile components using lattice structures for additive manufacturing. Mater. Test..

[2] Morciano M., Alberghini M., Fasano M., Almiento M., Calignano F., Manfredi D., Asinari P., Chiavazzo E. (2023). 3D printed lattice metal structures for enhanced heat transfer in latent heat storage systems. J. Energy Storage.

[3] Khosroshahi S.F., Tsampas S., Galvanetto U. (2018). Feasibility study on the use of a hierarchical lattice architecture for helmet liners. Mater. Today Commun..

[4] Nasim M., Hasan M.J., Galvanetto U. (2022). Impact behavior of energy absorbing helmet liners with PA12 lattice structures: A computational study. Int. J. Mech. Sci..

[5] Decker T., Kedziora S. (2024). Optimizing the Thickness of Functionally Graded Lattice Structures for High-Performance Energy Absorption: A Case Study Based on a Bicycle Helmet. Appl. Sci..

[6] Bazyar P., Sheidaee E. (2023). Design and simulating lattice structures in the FE analysis of the femur bone. Bioprinting.

[7] Seharing A., Azman A.H., Abdullah S. (2020). Finite element analysis of gradient lattice structure patterns for bone implant design. Int. J. Struct. Integr..

[8] Oladapo B.I., Ismail S.O., Ikumapayi O.M., Karagiannidis P.G. (2022). Impact of rGO-coated PEEK and lattice on bone implant. Colloids Surfaces B Biointerfaces.

[9] Ma X., Zhang D.Z., Zheng X. (2022). Revealing the excellent properties of minimal surface lattice structures based on additive manufacturing through the principle of least action. Int. J. Adv. Manuf. Technol..

[10] Wang W., Lin Y., Hu Y., Yang L., Lin D., Ma J., Zhou L., Liu B., Cai X., Yan C. (2025). The effect of fatigue loading on the mechanical properties of additively manufactured continuous carbon fiber-reinforced composites. Compos. Commun..

[11] Abou-Ali A.M., Lee D.W., Abu Al-Rub R.K. (2022). On the effect of lattice topology on mechanical properties of SLS additively manufactured sheet-, ligament-, and strut-based polymeric metamaterials. Polymers.

[12] Germain L., Fuentes C.A., van Vuure A.W., des Rieux A., Dupont-Gillain C. (2018). 3D-printed biodegradable gyroid scaffolds for tissue engineering applications. Mater. Des..

[13] Akbar S., Squires A.M., Elliott J.M. (2024). Production of a Metallic Single Gyroid with a Tunable Sub-10-nm Unit Cell Size via Lipid-Cubic-Phase Templating: Chiral Nanomaterials for Catalytic and Optical Applications. ACS Appl. Nano Mater..

[14] Su L., Lu F., Li Y., Wang Y., Li X., Zheng L., Gao X. (2024). Gyroid Liquid Crystals as Quasi-Solid-State Electrolytes Toward Ultrastable Zinc Batteries. ACS Nano.

[15] Lee J., Moon J., Han S.A., Kim J., Malgras V., Heo Y.U., Kim H., Lee S.M., Liu H.K., Dou S.X. (2019). Everlasting living and breathing gyroid 3D network in Si@ SiOx/C nanoarchitecture for lithium ion battery. ACS Nano.

[16] Choudhury S., Agrawal M., Formanek P., Jehnichen D., Fischer D., Krause B., Albrecht V., Stamm M., Ionov L. (2015). Nanoporous cathodes for high-energy Li–S batteries from gyroid block copolymer templates. ACS Nano.

[17] Zhang M., Hu T., Wang X., Chang P., Jin Z., Pan L., Mei H., Cheng L., Zhang L. (2022). Boosting uniform charge distribution using 3D rigid electrodes with interconnected gyroid channels to achieve stable and reliable zinc-ion batteries. J. Mater. Chem. A.

[18] Nguyen N.V., Tran K.Q., Do D.T., Thai C.H., Żur K.K., Nguyen-Xuan H. (2024). An isogeometric analysis of solar panels with a bio-inspired substrate. Eng. Anal. Bound. Elem..

[19] Wu S., Yang L., Yang X., Chen P., Su J., Wu H., Liu Z., Wang H., Wang C., Yan C. (2022). Mechanical properties and energy absorption of AlSi10Mg Gyroid lattice structures fabricated by selective laser melting. Smart Manuf..

[20] Zhang C., Zheng H., Yang L., Li Y., Jin J., Cao W., Yan C., Shi Y. (2022). Mechanical responses of sheet-based gyroid-type triply periodic minimal surface lattice structures fabricated using selective laser melting. Mater. Des..

[21] Du Plessis A., Razavi N., Berto F. (2020). The effects of microporosity in struts of gyroid lattice structures produced by laser powder bed fusion. Mater. Des..

[22] Jivrakh K.B., Varghese A.M., Ehrling S., Kuppireddy S., Polychronopoulou K., Al-Rub R.K.A., Alamoodi N., Karanikolos G.N. (2024). 3D-Printed zeolite 13X gyroid monolith adsorbents for CO_2_ capture. Chem. Eng. J..

[23] Ho J.Y., Chang T.T., Ho P.C., Chang H.K., Chen P.Y. (2024). Fabrication of gyroid-structured, hierarchically-porous hydroxyapatite scaffolds by a dual-templating method. Mater. Chem. Phys..

[24] Fina F., Goyanes A., Madla C.M., Awad A., Trenfield S.J., Kuek J.M., Patel P., Gaisford S., Basit A.W. (2018). 3D printing of drug-loaded gyroid lattices using selective laser sintering. Int. J. Pharm..

[25] Alizadeh-Osgouei M., Li Y., Vahid A., Ataee A., Wen C. (2021). High strength porous PLA gyroid scaffolds manufactured via fused deposition modeling for tissue-engineering applications. Smart Mater. Med..

[26] Salaha Z.F.M., Ammarullah M.I., Abdullah N.N.A.A., Aziz A.U.A., Gan H.S., Abdullah A.H., Abdul Kadir M.R., Ramlee M.H. (2023). Biomechanical effects of the porous structure of gyroid and voronoi hip implants: A finite element analysis using an experimentally validated model. Materials.

[27] Higuchi T. (1990). Relationship between the fractal dimension and the power law index for a time series: A numerical investigation. Phys. D Nonlinear Phenom..

[28] Beer T., Enting I.G. (1991). Fractals, lattice models, and environmental systems. Environ. Int..

[29] Gefen Y., Mandelbrot B.B., Aharony A. (1980). Critical phenomena on fractal lattices. Phys. Rev. Lett..

[30] Manna S., Nandy S., Roy B. (2022). Higher-order topological phases on fractal lattices. Phys. Rev. B.

[31] Kosior A., Sacha K. (2017). Localization in random fractal lattices. Phys. Rev. B.

[32] Zierenberg J., Fricke N., Marenz M., Spitzner F., Blavatska V., Janke W. (2017). Percolation thresholds and fractal dimensions for square and cubic lattices with long-range correlated defects. Phys. Rev. E.

[33] Zhang Z., Scarpa F., Bednarcyk B.A., Chen Y. (2021). Harnessing fractal cuts to design robust lattice metamaterials for energy dissipation. Addit. Manuf..

[34] Nguyen-Van V., Wu C., Vogel F., Zhang G., Nguyen-Xuan H., Tran P. (2021). Mechanical performance of fractal-like cementitious lightweight cellular structures: Numerical investigations. Compos. Struct..

[35] Rian I.M., Sassone M., Asayama S. (2018). From fractal geometry to architecture: Designing a grid-shell-like structure using the Takagi–Landsberg surface. Comput.-Aided Des..

[36] Cooper J.M. (2012). Introduction to Partial Differential Equations with MATLAB.

[37] Li J., Chen Y.T. (2019). Computational Partial Differential Equations Using MATLAB^®^.

[38] Pepper D.W., Heinrich J.C. (2017). The Finite Element Method: Basic Concepts and Applications with MATLAB, MAPLE, and COMSOL.

[39] Carpinteri A. (1994). Fractal nature of material microstructure and size effects on apparent mechanical properties. Mech. Mater..

[40] Xie H., Wang J.A., Xie W.H. (1997). Fractal effects of surface roughness on the mechanical behavior of rock joints. Chaos Solitons Fractals.

[41] Yang X., Dai H. (2020). Effect of geometric form of concrete meso-structure on its mechanical behavior under compression. Powder Technol..

[42] Sanchez-Molina D., Velazquez-Ameijide J., Quintana V., Arregui-Dalmases C., Crandall J.R., Subit D., Kerrigan J.R. (2013). Fractal dimension and mechanical properties of human cortical bone. Med Eng. Phys..

[43] Elías-Zúñiga A., Martínez-Romero O., Trejo D.O., Palacios-Pineda L.M. (2024). An Efficient Approach for Solving the Fractal, Damped Cubic-Quintic DUFFING’S Equation. Fractals.

[44] Elias-Zuniga A., Martinez-Romero O., Olvera-Trejo D., Perales-Martínez I.A., Palacios-Pineda L.M. (2024). Exploring Insects Free Flight: Enhancing the Dipteran Flight Model to Include Fractal Effects. Fractals.

[45] Chen R., Zhang W., Jia Y., Wang S., Cao B., Li C., Du J., Yu S., Wei J. (2024). Ultra-stiff and quasi-elastic-isotropic triply periodic minimal surface structures designed by deep learning. Mater. Des..

[46] Zhao M., Liu F., Fu G., Zhang D.Z., Zhang T., Zhou H. (2018). Improved mechanical properties and energy absorption of BCC lattice structures with triply periodic minimal surfaces fabricated by SLM. Materials.

[47] Lu J.Y., Silva T., Alzaabi F., Abu Al-Rub R.K., Lee D.W. (2024). Insights into acoustic properties of seven selected triply periodic minimal surfaces-based structures: A numerical study. J. Low Freq. Noise Vib. Act. Control.

[48] Fu H., Kaewunruen S. (2022). Experimental and DEM investigation of axially-loaded behaviours of IWP-based structures. Int. J. Mech. Sci..

[49] Elias-Zuniga A., Palacios-Pineda L.M., Jimenez-Cedeno I.H., Martinez-Romero O., Olvera-Trejo D. (2021). A fractal model for current generation in porous electrodes. J. Electroanal. Chem..

[50] Liu F., Mao Z., Zhang P., Zhang D.Z., Jiang J., Ma Z. (2018). Functionally graded porous scaffolds in multiple patterns: New design method, physical and mechanical properties. Mater. Des..

[51] Naghavi S.A., Tamaddon M., Marghoub A., Wang K., Babamiri B.B., Hazeli K., Xu W., Lu X., Sun C., Wang L. (2022). Mechanical characterisation and numerical modelling of TPMS-based gyroid and diamond Ti6Al4V scaffolds for bone implants: An integrated approach for translational consideration. Bioengineering.

[52] Maskery I., Aremu A.O., Parry L., Wildman R.D., Tuck C.J., Ashcroft I.A. (2018). Effective design and simulation of surface-based lattice structures featuring volume fraction and cell type grading. Mater. Des..

[53] Haney C.W., Siller H.R. (2023). Anthropo-fidelic behavior of elastic-plastic lattice structures. Polym. Test..

[54] De Groot R., Peters M., De Haan Y., Dop G., Plasschaert A. (1987). Failure stress criteria for composite resin. J. Dent. Res..

[55] Ausiello P., Rengo S., Davidson C.L., Watts D.C. (2004). Stress distributions in adhesively cemented ceramic and resin-composite Class II inlay restorations: A 3D-FEA study. Dent. Mater..

[56] Shim J.S., Watts D.C. (2000). An examination of the stress distribution in a soft-lined acrylic resin mandibular complete denture by finite element analysis. Int. J. Prosthodont..

[57] Ashok D., Bahubalendruni M.R., Mhaskar A., Choudhary V., Balamurali G., Turaka S. (2023). Experimental and numerical investigation on 2.5-dimensional nature-inspired infill structures under out-plane quasi-static loading. Proc. Inst. Mech. Eng. Part E J. Process Mech. Eng..

